# A 3-year school-based exercise intervention improves muscle strength - a prospective controlled population-based study in 223 children

**DOI:** 10.1186/1471-2474-15-353

**Published:** 2014-10-27

**Authors:** Fredrik Detter, Jan-Åke Nilsson, Caroline Karlsson, Magnus Dencker, Björn E Rosengren, Magnus K Karlsson

**Affiliations:** Clinical and Molecular Osteoporosis Research Unit, Department of Orthopedics and Clinical Sciences, Skåne University Hospital, SE-205 02 Malmö, Sweden; Department of Physiology, Lund University, Skåne University Hospital, SE-205 02 Malmö, Sweden

**Keywords:** Body composition, Boys, Isokinetic peak torque, Girls, Knee extension, Knee flexion, Muscle strength, Physical activity, School-based intervention

## Abstract

**Background:**

Intense physical activity (PA) improves muscle strength in children, but it remains uncertain whether moderately intense PA in a population-based cohort of children confers these benefits.

**Methods:**

We included children aged 6–9 years in four schools where the intervention school increased the school curriculum of PA from 60 minutes/week to 40 minutes/school day while the control schools continued with 60 minutes/week for three years. We measured muscle strength, as isokinetic Peak Torque (PT) (Nm) of the knee flexors in the right leg at speeds of 60°/second and 180°/second, at baseline and at follow-up, in 47 girls and 76 boys in the intervention group and 46 girls and 54 boys in the control group and then calculated annual changes in muscle strength. Data are provided as means with 95% confidence intervals.

**Results:**

Girls in the intervention group had 1.0 Nm (0.13, 1.9) and boys 1.9 Nm (0.9, 2.9) greater annual gain in knee flexor PT at 60**°/** second, than girls and boys in the control group. Boys in the intervention group also had 1.5 Nm (0.5, 2.5) greater annual gain in knee flexors PT at 180**°/** second than boys in the control group.

**Conclusion:**

A 3-year moderately intense PA intervention program within the school curriculum enhances muscle strength in both girls and boys.

**Electronic supplementary material:**

The online version of this article (doi:10.1186/1471-2474-15-353) contains supplementary material, which is available to authorized users.

## Background

In the context of a growing and aging population, preventive strategies are needed for diseases of old age, including falls and fragility fractures. Physical activity (PA) could be one such strategy since PA in childhood is associated with improved aerobic fitness, bone mass, muscle strength, muscle function and fat profile [1–5]. Whether or not the response to PA is similar in boys and girls is unclear since there are gender differences in the response to PA [6–8]. Furthermore, the timing of an increase in the level of PA has been disputed since the musculoskeletal complex may, like the skeleton, respond differently at different pubertal stages [1, 6, 7, 9–14]. The skeleton responds most favorably to mechanical load during the late pre- and early pubertal period [3, 5, 13, 15, 16], but whether or not muscles show a similar pattern is debated [1, 6–8, 14, 17–19]. Some reports indicate that the pubertal growth spurt and the transient increased accrual of bone mineral are not accompanied by a pubertal boost in muscle strength [20]. The divergent inferences probably reflect the fact that the studies included different proportions of girls and boys, evaluated children in different ages and with different pubertal maturation and also used different training protocols [1, 6–8, 14, 17–19].

Generalized international guidelines currently recommend 60 minutes’ daily moderate activity with inclusion of vigorous activity on at least three days/week [4]. It is unknown whether the same level should be advocated in children and if so, whether the same level should be recommended in all ages. Furthermore, there is probably a need for different types and intensities of PA when gain in muscle strength, bone mass or cardiovascular health are used as endpoint variables. Furthermore, a PA intervention program should include a variety of activities, since specific designed monotonous training programs usually result in a large dropout frequency [3, 11].

The aim of this study was to determine whether a 3-year school-based PA intervention program affected the gain in muscle strength and lean body mass (muscle mass) in children, who were pre-pubertal at study start.

## Methods

### Study design

The Paediatric Osteoporosis Prevention (POP) study is a prospective controlled exercise intervention trial that evaluates musculoskeletal development in children aged 6–9 years at study start. The study design and methodology have previously been described in detail [6, 8, 21–27], was approved by the Ethics Committee at Lund University and the study was conducted in accordance with the Declaration of Helsinki. We obtained written informed consent from parents or guardians and children prior to inclusion. To summarize the study design, we included four community-based schools within the same geographic area where children were allocated to school according to residential address. The cohort could therefore be regarded as a cluster of convenience, the schools being the clusters and the convenience that they are all from the same neighborhood. One school was assigned as intervention school and three as control schools. In the intervention school we increased the amount of physical education (PE) in the school curriculum from 60 minutes PE/week to 40 minutes/school day (200 minutes per week) for three years. The intervention consisted of a variety of activities such as jumping, running, playing and ball games, i.e. the regular Swedish school curriculum for PE but with an extended duration [6, 8, 21–27]. The control schools used the same type of PE but continued with the duration of 60 minutes/week [6, 8, 21–27]. The ordinary teachers supervised the PE classes. We provided no extra PA during vacation and weekends.

### Study material

**Figure 1:**
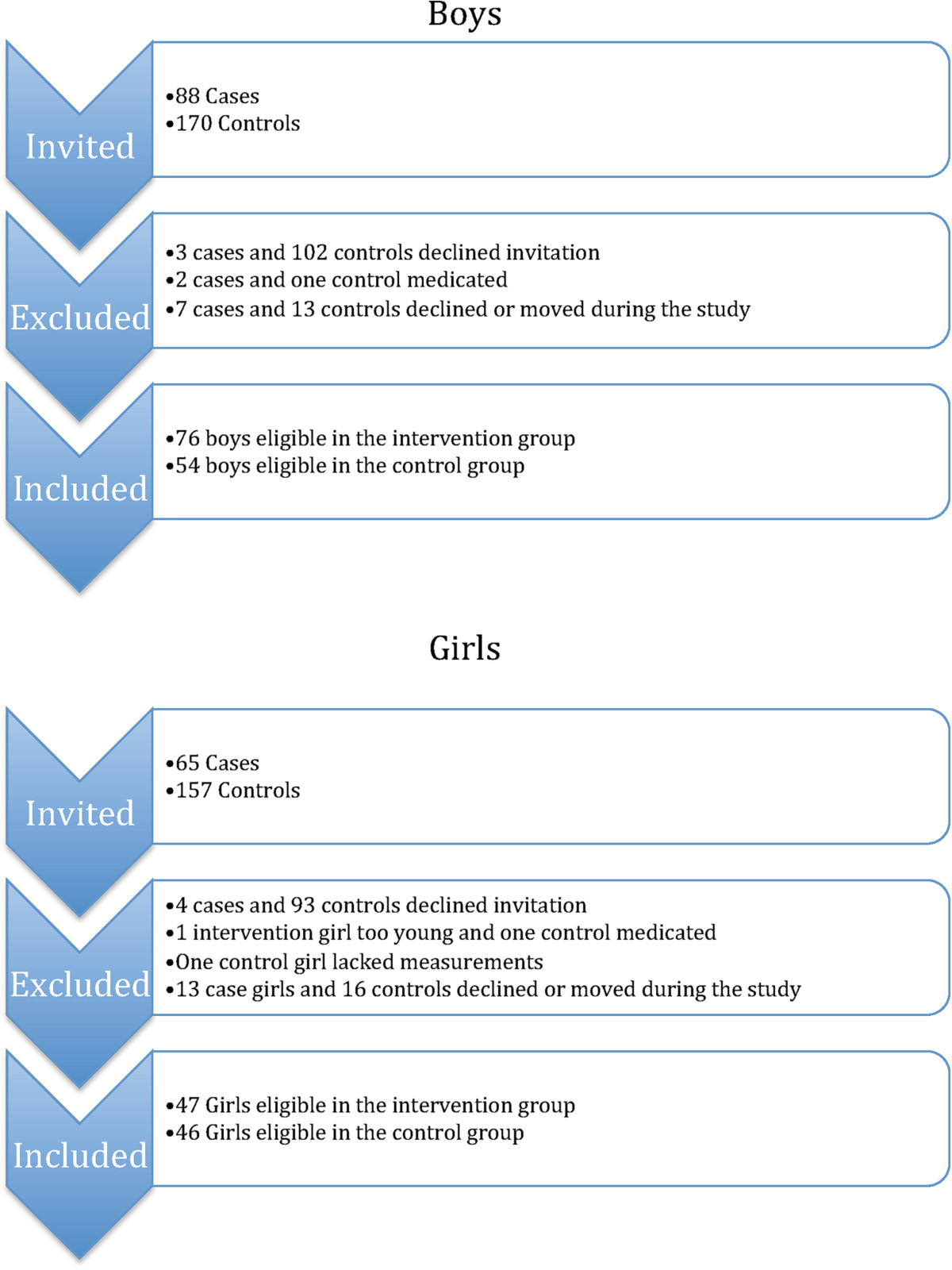
**Flow-chart of the study participants.**

We invited all children who started school during two consecutive years to participate in repeated measurements of anthropometry, muscle strength and body composition. Figure 1 shows a flow-chart of the participants in this study. Out of the 65 invited girls in the intervention school, 61 agreed to participate at baseline while 47 subjects continued throughout the study with the inclusion criteria fulfilled (Figure 1). The corresponding figure in the intervention boys was 88 invited, 85 with baseline measurements and 76 with prospective data (Figure 1). In the control group 157 girls and 170 boys were invited, 64 girls and 68 boys participated in the baseline measurements and 46 girls and 54 boys had prospective data (Figure 1). The children were 6–9 years old at baseline and 10–12 at follow-up. A dropout analysis showed that there were no differences in height, weight, body mass index (BMI), total body or regional body composition or muscle strength between children who took part in both baseline and follow-up visits and those that only attended baseline. In the grade one compulsory school health examination we found similar age, height, weight and BMI in the children who participated in the baseline measurements and those who declined participation [6, 8, 21–27].

### Muscular strength

Two physiotherapists measured muscle strength by a computerized dynamometer (Biodex System 3®) as concentric isokinetic Peak Torque (PT) of the right knee extensors (ex) and flexors (fl) at speeds of 60 and 180°/second (°/sec). Study participants were seated with their hips at 85° flexion from anatomical position during the testing. The knee axis was aligned with the axis of rotation of the Biodex dynamometer. Study participants were fastened according to standard procedure using the Biodex machine utilizing shin, thigh, pelvic and upper torso crossing stabilization straps. When the lumbar lordosis created a space between the participant’s back and the chair, a 10 cm thick pad was used to fill the space. If the lever of the Biodex machine was longer than the lower leg we used a pad to adjust the difference. All participants were instructed to cross their arms on the chest during the test. During the testing, the knee joint went through a 75° range of motion between 20° and 95° flexion. Before each test the children performed three sub-maximal practice repetitions in order to get familiar with the machine and the movements and the children received visual and verbal encouragement during the testing. The test included five 60°/sec repetitions (flexion and extension) followed by 30 seconds of rest and then ten 180°/sec repetitions (flexion and extension), all at maximal effort. The highest recorded peak torque (Nm) value for each setup was registered.

Peak torque was then normalized to weight (Nm/kg) in all measurements. The precision of the measurements evaluated as coefficients of variation (CV) in 21 children was 6.6% for PT_Ex60_, 12.1% for PT_Fl60_, 12.3% for PT_Ex180_ and 9.1% for PT_Fl180_. 58-.

### Anthropometry and body composition

We measured weight with an Avery Berkel HL 120 Electric Scale® and height with a Holtain Stadiometer® and calculated BMI as weight/height^2^ (kg/m^2^). We measured total body and regional fat and lean mass by dual energy X-ray absorptiometry (DXA) (DPX-L version 1.3z, Lunar®) in a total body scan. Our research technicians performed and analyzed all scans. The intra-individual test variability (CV%) was 3.7% for total body fat and 1.5% for total body lean mass, assessed after repeated measurements in 13 healthy children.

### Lifestyle

We used a lifestyle questionnaire, utilized in several previous studies [6, 8, 21–27], to evaluate nutrition, diseases, medications, PA and lifestyle at baseline and follow-up (Table 1). We evaluated PA separately for activities taking place within the school curriculum and for leisure time organized PA (Table 2). Our research nurse instructed the children to self-assess Tanner stage [28] at baseline and at follow-up.

**Table 1:** **Lifestyle factors**

	Girls			Boys		
	Cases (n = 47)	Controls (n = 46)	***P-*** value	Cases (n = 76)	Controls (n = 54)	***P-*** value
**Baseline**						
**Age**	7.7 (0.6)	7.9 (0.6)	0.07	7.8 (0.6)	7.9 (0.6)	0.23
**Lifestyle factors**						
Excluding dairy products	0	3 (7%)	0.11	1 (1%)	7 (13%)	**<0.01**
Drinking coffee	3 (6%)	1 (2%)	0.62	3 (4%)	0	0.27
Smoking	0	0	NA	0	0	NA
Alcohol	0	0	NA	0	0	NA
Tried to lose weight	1 (2%)	0	1.0	0	0	NA
Current disease	3 (6%)	3 (7%)	1.0	7 (9%)	3 (6%)	0.52
Ongoing medication	5 (11%)	2 (4%)	0.43	10 (13%)	4 (7%)	0.39
Previous medication	4 (9%)	2 (4%)	0.68	3 (4%)	5 (9%)	0.28
Previous Fracture	5 (11%)	6 (13%)	0.72	6 (8%)	6 (11%)	0.53
Tanner stage 1/2/3/4/5	47/0/0/0/0	46/0/0/0/0	NA	76/0/0/0/0	48/0/0/0/0	NA
**Follow-up (after 3 years)**						
**Age**	10.7 (0.6)	11.1 (0.7)	**0.003**	10.8 (0.6)	11.1 (0.6)	**0.01**
**Lifestyle factors**						
Smoking	0	0	NA	0	0	NA
Alcohol	0	0	NA	0	0	NA
Tanner stage 1/2/3/4/5 (%)	17/18/9/2/0	10/20/14/2/0	0.12	65/9/2/0/0	11/26/14/1/0	**<0.001**
Menarche	3 (6%)	1 (2%)	0.62	——	——	——

Baseline and follow-up data in the subsample of girls and boys who were measured, presented as numbers and proportion (%) or as means with standard deviations (SD). Statistically significant differences are bolded.

**Table 2:** **Physical activity**

	Girls			Boys		
	Cases (n = 47)	Controls (n = 46)	***P-*** value	Cases (n = 76)	Controls (n = 54)	***P-*** value
**Baseline**						
**Organized physical activity before study start (hours/week)**						
Total physical activity	1.7 (0.7)	2.4 (1.7)	**0.02**	2.7 (1.6)	2.3 (1.3)	0.18
**Organized physical activity after study start (hours/week)**						
School curriculum	3.3	1.0	**<0.001**	3.3	1.0	**<0.001**
Outside School	0.7 (0.7)	1.4 (1.7)	**0.02**	1.7 (1.6)	1.3 (1.3)	0.18
Total physical activity	4.0 (0.7)	2.4 (1.7)	**<0.001**	5.0 (1.6)	2.3 (1.3)	**<0.001**
**Follow-up (after 3 years)**						
**Organized physical activity (hours/week)**						
School curriculum	3.3	1.0	**<0.001**	3.3	1.0	**<0.001**
Outside School	2.3 (2.0)	2.8 (2.5)	0.38	3.3 (3.3)	2.9 (2.4)	0.50
Total physical activity	5.6 (2.0)	3.8 (2.5)	**<0.001**	6.6 (3.3)	3.9 (2.4)	**<0.001**

Baseline and follow-up physical activity data in the subsample of girls and boys who were measured. Questionnaire-evaluated duration of organized physical activity was estimated as mean hours per week. Data are presented as mean with standard deviation (SD). Statistically significant group differences are bolded.

### Statistical analysis

We used IBM SPSS Statistics® version 20 to perform statistical analyses. Values that were biologically unlikely, defined as above or below three standard deviations (SD) from the mean, were excluded, as described by Beck et al. [29]. This resulted in the exclusion of 16 dynamometer, anthropometry or DXA measurements. Background data are presented as proportions, means ± standard deviations (SD) or means with ranges and results as means with 95% confidence intervals (95% CI). Levene’s test was utilized to test homoscedasticity. Annual changes were calculated as the difference between the baseline and follow-up measurements divided by follow-up time. Gender-specific group differences were evaluated by student’s *t*-test between means, chi-squared test, Fisher’s exact test or Mann–Whitney *U*-test depending on the analysis. We used ANCOVA to adjust for group differences in age, annual change in height and Tanner-stage at follow-up. We regarded a p < 0.05 as a statistically significant difference. A post-hoc power analysis revealed that we had 80% power to detect a difference of 2.3 Nm in PTex60, 1.2 Nm in PTfl60 and 0.1 kg in lean mass in boys and of 2.1 Nm in PTex60, 1.3 Nm in PTfl60 and 0.2 kg in lean mass in girls with a significance level of 0.05.

## Results

The only registered lifestyle discrepancy was that more boys in the control group excluded dairy products than boys in the intervention group (Table 1). Before the intervention was initiated, PA was similar in subjects and controls (Table 2). After the intervention was initiated, both girls and boys in the intervention group reported significantly higher duration of total PA compared to controls (Table 2). At baseline both boys and girls in the intervention group had lower muscle strength than boys and girls in the control group (Table 3).

**Table 3:** **Muscle strength**

Girls										
	Baseline		Between-group differences baseline			Annual changes		Between-group differences annual changes		
	Cases (n = 47)	Controls (n = 46)	Adjusted difference (95% CI)	P_1_-value	P_2_-value	Cases (n = 47)	Controls (n = 46)	Adjusted difference (95% CI)	P_3_-value	P_4_-value
**Anthropometry (kg)**										
Height (cm)	128.0 (5.2)	129.9 (7.9)	-0.5 (-2.8, 1.9)	0.18	0.70	6.2 (5.9, 6.5)	6.0 (5.7, 6.3)	0.3 (-0.1, 0.03)	0.48	0.09
Weight (kg)	27.6 (5.5)	27.8 (5.6)	-0.2 (-2.1, 2.5)	0.90	0.84	4.1 (3.6, 4.5)	3.5 (3.1, 3.9)	0.4 (-0.01, 0.8)	**0.04**	**0.05**
BMI (kg/m^2^)	16.8 (3.0)	16.3 (1.9)	0.4 (-0.7, 1.4)	0.35	0.48	0.6 (0.4, 0.7)	0.4 (0.2, 0.5)	0.2 (0.01, 0.3)	**0.02**	**0.04**
**Lean body mass (kg)**										
Total Body	19.8 (2.3)	20.4 (2.8)	-0.2 (-1.2, 0.8)	0.25	0.66	2.4 (2.2, 2.6)	2.3 (2.1, 2.5)	0.1 (-0.03, 0.3)	0.37	0.12
Legs	6.3 (1.0)	6.4 (1.2)	-0.03 (0.4, 0.5)	0.55	0.87	1.0 (0.9, 1.1)	1.0 (0.9, 1.0)	0.04 (-0.03, 0.1)	0.37	0.24
Arms	1.6 (0.3)	1.7 (0.4)	-0.1 (-0.2, 0.1)	0.10	0.20	0.3 (0.2, 0.3)	0.2 (0.2, 0.2)	0.04 (-0.01, 0.1)	0.10	**0.01**
**Fat mass (kg)**										
Total Body	4.8 (3.1)	5.0 (3.1)	0.2 (-1.5, 1.1)	0.76	0.76	1.6 (1.3, 1.9)	1.0 (0.7, 1.2)	0.5 (0.2, 0.9)	**0.003**	**0.001**
Legs	2.1 (1.1)	2.2 (1.2)	0.1 (-0.6, 0.4)	0.70	0.73	0.6 (0.5, 0.7)	0.4 (0.3, 0.5)	0.2 (0.1, 0.3)	**0.02**	**0.002**
Arms	0.5 (0.4)	0.5 (0.4)	-0.04 (-0.2, 0.1)	0.68	0.66	0.1 (0.1, 0.2)	0.1 (0.03, 0.1)	0.1 (0.02, 0.1)	**<0.001**	**0.03**
**Peak torque (Nm)**										
PT_Ex60_	40.9 (9.7)	44.4 (9.8)	-1.6 (-5.2, 1.9)	0.09	0.36	10.5 (9.3, 11.8)	10.8 (9.7, 12.0)	-0.6 (-2.1, 0.8)	0.75	0.40
PT_Fl60_	19.6 (5.1)	24.4 (6.2)	-4.1 (-6.4, -1.9)	**<0.001**	**<0.001**	6.3 (5.7, 7.0)	5.2 (4.6, 5.9)	1.0 (0.1, 1.9)	**0.02**	**0.03**
PT_Ex180_	31.9 (7.9)	36.1 (7.4)	-2.8 (-5.7, 0.1)	**0.01**	0.06	7.4 (6.6, 8.2)	7.1 (6.4, 7.7)	0.3 (-0.6, 1.2)	0.54	0.57
PT_Fl180_	16.9 (5.5)	22.1 (4.6)	-4.5 (-6.5, -2.5)	**<0.001**	**<0.001**	4.8 (4.2, 5.4)	4.1 (3.4, 4.7)	0.4 (0.5, 1.4)	0.10	0.35
**Relative peak torque (Nm/Kg)**										
PT_Ex60_	1.5 (0.3)	1.7 (0.3)	-0.1 (-0.2, 0.03)	**0.03**	0.13	0.1 (0.1, 0.2)	0.1 (0.1, 0.2)	-3.4 (-6.9, 0.2)	0.26	0.06
PT_Fl60_	0.7 (0.2)	0.9 (0.1)	-0.2 (-0.2, -0.1)	**<0.001**	**<0.001**	0.1 (0.1, 0.1)	0.1 (0.04, 0.1)	1.5 (-1.3, 4.3)	0.08	0.30
PT_Ex180_	1.2 (0.2)	1.3 (0.2)	-0.1 (-0.2. -0.03)	**0.001**	**<0.01**	0.1 (0.1, 0.1)	0.1 (0.1, 0.1)	-1.0 (-3.2, 1.3)	0.91	0.41
PT_Fl180_	0.6 (0.2)	0.8 (0.1)	-0.1 (-0.2, -0.1)	**<0.001**	**<0.001**	0.1 (0.04, 0.1)	0.03 (0.02, 0.1)	-1.0 (-3.4, 1.5)	**0.048**	0.44
**Boys**										
	**Baseline**		**Between-group differences baseline**			**Annual changes**		**Between-group differences annual changes**		
	**Cases (n = 76)**	**Controls (n = 54)**	**Adjusted difference (95% CI)**	**P**_**1**_ **-Value**	**P**_**2**_ **-Value**	**Cases (n = 76)**	**Controls (n = 54)**	**Adjusted difference (95% CI)**	**P**_**3**_ **-Value**	**P**_**4**_ **-Value**
**Anthropometry (kg)**										
Height (cm)	129.4 (6.6)	130.0 (6.8)	-0.2 (-1.8, 2.2)	0.62	0.83	5.5 (5.3, 5.6)	5.6 (5.4, 5.8)	0.1 (-0.2, 0.4)	0.32	0.53
Weight (kg)	28.4 (5.8)	27.8 (5.6)	1.2 (-0.6, 3.0)	0.54	0.20	3.5 (3.2, 3.8)	3.6 (3.3, 3.9)	-0.004 (-0.5, 0.4)	0.71	0.88
BMI (kg/m^2^)	16.8 (2.5)	16.3 (2.1)	0.7 (-0.1, 1.5)	0.21	0.11	0.4 (0.3, 0.5)	0.5 (0.4, 0.6)	-0.1 (-0.3, 0.1)	0.30	0.32
**Lean body mass (kg)**										
Total Body	21.8 (3.1)	21.6 (2.9)	0.5 (-0.4, 1.4)	0.72	0.27	2.2 (2.1, 2.3)	2.3 (2.2, 2.4)	-0.1 (-0.2, 0.1)	0.28	0.60
Legs	6.8 (1.3)	6.8 (1.3)	0.2 (-0.2, 0.6)	0.81	0.27	1.0 (0.9, 1.0)	1.1 (1.0, 1.1)	-0.1 (-0.1, -0.02)	0.05	0.16
Arms	1.8 (0.4)	1.8 (0.4)	0.04 (-0.1, 0.2)	0.90	0.47	0.3 (0.2, 0.3)	0.2 (0.2, 0.2)	0.03 (-0.004, 0.1)	0.16	0.08
**Fat mass (kg)**										
Total Body	3.8 (3.0)	3.7 (2.6)	0.3 (-0.7, 1.3)	0.81	0.56	1.1 (0.9, 1.4)	1.1 (0.8, 1.3)	0.1 (-0.2, 0.4)	0.62	0.57
Legs	1.7 (1.1)	1.6 (0.9)	0.1 (-0.2, 0.5)	0.66	0.40	0.5 (0.4, 0.6)	0.4 (0.4, 0.5)	0.01 (-0.1, 0.1)	0.64	0.90
Arms	0.3 (0.3)	0.4 (0.4)	0.1 (-0.2, 0.1)	0.27	0.41	0.1 (0.1, 0.1))	0.1 (0.03, 0.1)	0.03 (-0.01, 0.1)	**0.03**	0.11
**Peak torque (Nm)**										
PT_Ex60_	43.9 (11.0)	44.6 (10.9)	0.6 (-2.5, 3.7)	0.71	0.70	9.8 (8.8, 10.7)	10.8 (9.6, 12.0)	-0.8 (-2.5, 0.9)	0.17	0.36
PT_Fl60_	21.8 (6.7)	25.0 (6.7)	-2.4 (-4.5, -0.4)	**0.01**	**0.02**	7.1 (6.6, 7.7)	5.9 (5.3, 6.5)	1.9 (0.9, 2.9)	**0.005**	**<0.001**
PT_Ex180_	35.9 (8.3)	35.8 (7.5)	1.1 (-1.1, 3.3)	0.90	0.34	7.0 (6.5, 7.5)	7.6 (7.0, 8.3)	-0.4 (-1.3, 0.5)	0.15	0.42
PT_Fl180_	19.3 (5.5)	23.0 (5.1)	-3.2 (-4.9, -1.6)	**<0.001**	**<0.001**	5.6 (5.1, 6.1)	4.3 (3.8, 4.8)	1.5 (0.5, 2.5)	**0.001**	**0.004**
**Relative peak torque (Nm/Kg)**										
PT_Ex60_	1.6 (0.2)	1.7 (0.3)	-0.1 (-0.2, 0.01)	**<0.05**	0.10	0.1 (0.1, 0.1)	0.1 (0.1, 0.2)	-0.5 (-4.6, 3.6)	0.82	0.80
PT_Fl60_	0.8 (0.2)	0.9 (0.2)	-0.1 (-0.2, -0.1)	**<0.001**	**<0.001**	0.1 (0.1, 0.1)	0.1 (0.06, 0.1)	3.2 (0.6, 5.8)	**<0.001**	**0.02**
PT_Ex180_	1.3 (0.2)	1.3 (0.2)	-0.1 (-0.1, 0.02)	0.07	0.15	0.1 (0.1, 0.1)	0.1 (0.1, 0.01)	0.8 (-3.2, 1.6)	0.62	0.51
PT_Fl180_	0.7 (0.2)	0.9 (0.1)	-0.2 (-0.2, -0.1)	**<0.001**	**<0.001**	0.1 (0.1, 0.1)	0.03 (0.03, 0.1)	3.1 (0.7, 5.5)	**<0.001**	**0.01**

Baseline data and annual changes in the subsample of girls and boys who were measured. Baseline data are presented as unadjusted means with standard deviation (SD). Group comparisons of baseline data were done unadjusted (p_1_) and adjusted for age at baseline (p_2_). Annual changes are presented as unadjusted means with 95% confidence intervals (95% CI). Mean difference, in annual changes, are presented as adjusted mean (95% CI). Group comparison of annual changes is done unadjusted (p_3_) and (p_4_) adjusted for age at baseline, baseline parameter, annual change in height and Tanner stage at follow-up. Statistically significant changes are bolded.

The annual increase in PT flexion strength was significantly greater in both girls and boys in the intervention group than in controls (p <0.01 to <0.05) (Table 3). The adjusted annual gain in lean mass arms was greater in intervention girls than controls, as was the gain in fat mass (Table 3).

## Discussion

In this three-year prospective controlled population-based study in pre-pubertal children we found that intervention with moderately intense PA conferred a greater gain in muscle strength in both girls and boys. The study provides high-level of evidence that supports daily PE in the school curriculum as a strategy to improve muscle strength in the general pediatric population. This has important public health implications since low muscle strength and neuromuscular function are associated with falls and fractures [2, 30] and high muscular strength with good balance, good postural control, and lower fall and fracture risks [31–33].

Current literature suggests that training improves muscular function [7, 8, 14, 19], and specific intervention programs are reported to confer excellent effects on musculoskeletal performance [3, 18, 34–36]. But studies on PA-induced muscular effects in pre-pubertal children are rare and the results are conflicting [6, 8, 14, 19, 37–39]. This could be explained by the fact that the studies have used different techniques to assess muscle strength and have included heterogeneous cohorts with respect to age, pubertal maturation, height, weight and BMI, all traits influencing muscle strength [6, 8, 14, 19, 37–39]. Furthermore, since all prospective studies are short-term, little is known about long-term effects of PA intervention. Most studies also use volunteers with an interest in exercise, who are thus probably easier to motivate to participate in PA, while few studies have a population-based design. This study should therefore not be considered as another study that explores the effect of specific training modalities but a study that shows that increased PA within the school curriculum could be used as a strategy to improve muscle strength on the population level.

In this study we found benefits in muscle strength in both boys and girls. When evaluating lean body mass (muscle mass), we found benefits only in the arms in the girls. A large muscle mass may improve muscle strength [36, 40–43]. However, the gain in muscle strength in children may also be explained by neural adaptations such as complex influences in neuromuscular interaction in the motor unit and increased coordination of agonists and antagonists [7, 14, 17, 42, 44]. In other words, a child could gain muscle strength without increasing muscle mass. In our study the reason for increased muscle strength, as measured in the lower extremities with no increased lean body mass, ought to depend on motor unit activation, coordination, recruitment, and/or firing frequency [2, 14, 44].

The duration of physical activity necessary to gain muscular benefits during growth is not defined, but international guidelines recommend 60 minutes of varied physical activity per day [3, 4, 45] with the inclusion of vigorous exercises for at least 3 days per week [46]. But it is unknown whether this recommendation also accounts for children and all traits such as muscle strength, bone mass or cardiovascular health. Our study results indicate that the effects achieved by this level of PA are beneficial. Whether or not even greater benefits can be reached by a higher level of physical activity ought to be evaluated in future trials. Another important aspect is that if children change a sedentary lifestyle to a more active one early in childhood, they are more likely to continue with a healthy and active lifestyle in adult life as well [47, 48].

We also found a larger gain in fat mass in the girls with extra PA than in the control group. The reason for the larger fat mass gain in girls remains unknown, contrasting with the fact that a high level of PA in most studies is associated with low fat content [1–5]. We speculate that with increased PA, there is also an increase in food intake. We have previously shown a lack of a dose–response relationship between duration of PA and gain in fat mass [8, 23] and also a higher transient fat gain in boys [22]. With this in mind, the group difference in fat mass gain in girls is probably the result of chance.

The study strengths include the population-based design, the high participation rate, and the thorough dropout analyses. The study is the prospective controlled PA intervention study with muscle strength and lean mass as endpoint variables with the longest duration so far, to our knowledge. Limitations include the lack of individual randomization, a design not practically feasible due to the school organization, the lower participation rate in control schools, and the lack of registration of non-organized PA during spare time and vacations. Another weakness is the lack of leg length measurements, as leg length is known to affect the outcome of knee extension and flexion PT strength due to differences in muscle moment arm [49]. We addressed this concern as best we could by adjusting for differences in annual height change.

## Conclusion

A three-year physical activity intervention program in pre-pubertal children improves muscular strength. More school physical education should be implemented to improve muscle strength, as this seems a feasible strategy to reduce the number of fall and fractures in the long term.

## Authors’ original submitted files for images

Below are the links to the authors’ original submitted files for images. Authors’ original file for figure 1 Authors’ original file for figure 2

## References

[1] Ara I, Vicente-Rodriguez G, Jimenez-Ramirez J, Dorado C, Serrano-Sanchez JA, Calbet JA (2004). Regular participation in sports is associated with enhanced physical fitness and lower fat mass in prepubertal boys. Int J Obes Relat Metab Disord.

[2] Behm DG, Faigenbaum AD, Falk B, Klentrou P (2008). Canadian Society for Exercise Physiology position paper: resistance training in children and adolescents. Appl Physiol Nutr Metab.

[3] Boreham CA, McKay HA (2011). Physical activity in childhood and bone health. Br J Sports Med.

[4] Anderson J, Parker W, Steyn NP, Grimsrud A, Kolbe-Alexander T, Lambert EV, Mciza Z (2009). In Interventions on Diet and Physical Activity: What Works: Summary Report.

[5] Bailey DA, McKay HA, Mirwald RL, Crocker PR, Faulkner RA (1999). A six-year longitudinal study of the relationship of physical activity to bone mineral accrual in growing children: the university of Saskatchewan bone mineral accrual study. J Bone Miner Res Off J Am Soc Bone Miner Res.

[6] Stenevi-Lundgren S, Daly RM, Karlsson MK (2010). A school-based exercise intervention program increases muscle strength in prepubertal boys. Int J Pediatr.

[7] Weltman A, Janney C, Rians CB, Strand K, Berg B, Tippitt S, Wise J, Cahill BR, Katch FI (1986). The effects of hydraulic resistance strength training in pre-pubertal males. Med Sci Sports Exerc.

[8] Stenevi-Lundgren S, Daly RM, Linden C, Gardsell P, Karlsson MK (2009). Effects of a daily school based physical activity intervention program on muscle development in prepubertal girls. Eur J Appl Physiol.

[9] De Ste Croix MB, Deighan MA, Ratel S, Armstrong N (2009). Age- and sex-associated differences in isokinetic knee muscle endurance between young children and adults. Appl Physiol Nutr Metab.

[10] Baxter-Jones AD, Eisenmann JC, Mirwald RL, Faulkner RA, Bailey DA (2008). The influence of physical activity on lean mass accrual during adolescence: a longitudinal analysis. J Appl Physiol (1985).

[11] MacKelvie KJ, Khan KM, Petit MA, Janssen PA, McKay HA (2003). A school-based exercise intervention elicits substantial bone health benefits: a 2-year randomized controlled trial in girls. Pediatrics.

[12] Karlsson MK, Linden C, Karlsson C, Johnell O, Obrant K, Seeman E (2000). Exercise during growth and bone mineral density and fractures in old age. Lancet.

[13] Kannus P, Haapasalo H, Sankelo M, Sievanen H, Pasanen M, Heinonen A, Oja P, Vuori I (1995). Effect of starting age of physical activity on bone mass in the dominant arm of tennis and squash players. Ann Intern Med.

[14] Ramsay JA, Blimkie CJ, Smith K, Garner S, MacDougall JD, Sale DG (1990). Strength training effects in prepubescent boys. Med Sci Sports Exerc.

[15] Bielemann RM, Martinez-Mesa J, Gigante DP (2013). Physical activity during life course and bone mass: a systematic review of methods and findings from cohort studies with young adults. BMC Musculoskelet Disord.

[16] Bass S, Pearce G, Bradney M, Hendrich E, Delmas PD, Harding A, Seeman E (1998). Exercise before puberty may confer residual benefits in bone density in adulthood: studies in active prepubertal and retired female gymnasts. J Bone Miner Res Off J Am Soc Bone Miner Res.

[17] Ara I, Vicente-Rodriguez G, Perez-Gomez J, Jimenez-Ramirez J, Serrano-Sanchez JA, Dorado C, Calbet JA (2006). Influence of extracurricular sport activities on body composition and physical fitness in boys: a 3-year longitudinal study. Int J Obes (Lond).

[18] Faigenbaum AD, Kraemer WJ, Blimkie CJ, Jeffreys I, Micheli LJ, Nitka M, Rowland TW (2009). Youth resistance training: updated position statement paper from the national strength and conditioning association. J Strength Cond Res.

[19] Sollerhed AC, Ejlertsson G (2008). Physical benefits of expanded physical education in primary school: findings from a 3-year intervention study in Sweden. Scand J Med Sci Sports.

[20] Behringer M, Vom Heede A, Yue Z, Mester J (2010). Effects of resistance training in children and adolescents: a meta-analysis. Pediatrics.

[21] Detter F, Rosengren BE, Dencker M, Lorentzon M, Nilsson JA, Karlsson MK (2014). A Six-year exercise program improves skeletal traits without affecting fracture risk - a prospective controlled study in 2621 children. J Bone Miner Res.

[22] Detter FT, Rosengren BE, Dencker M, Nilsson JA, Karlsson MK (2013). A 5-year exercise program in pre- and peripubertal children improves bone mass and bone size without affecting fracture risk. Calcif Tissue Int.

[23] Linden C, Alwis G, Ahlborg H, Gardsell P, Valdimarsson O, Stenevi-Lundgren S, Besjakov J, Karlsson MK (2007). Exercise, bone mass and bone size in prepubertal boys: one-year data from the pediatric osteoporosis prevention study. Scand J Med Sci Sports.

[24] Lofgren B, Dencker M, Nilsson JA, Karlsson MK (2012). A 4-year exercise program in children increases bone mass without increasing fracture risk. Pediatrics.

[25] Lofgren B, Detter F, Dencker M, Stenevi-Lundgren S, Nilsson JA, Karlsson MK (2011). Influence of a 3-year exercise intervention program on fracture risk, bone mass, and bone size in prepubertal children. J Bone Miner Res Off J Am Soc Bone Miner Res.

[26] Lofgren B, Stenevi-Lundgren S, Dencker M, Karlsson MK (2010). The mode of school transportation in pre-pubertal children does not influence the accrual of bone mineral or the gain in bone size–two year prospective data from the paediatric osteoporosis preventive (POP) study. BMC Musculoskelet Disord.

[27] Valdimarsson O, Linden C, Johnell O, Gardsell P, Karlsson MK (2006). Daily physical education in the school curriculum in prepubertal girls during 1 year is followed by an increase in bone mineral accrual and bone width–data from the prospective controlled Malmo pediatric osteoporosis prevention study. Calcif Tissue Int.

[28] Duke PM, Litt IF, Gross RT (1980). Adolescents’ self-assessment of sexual maturation. Pediatrics.

[29] Beck TJ, Oreskovic TL, Stone KL, Ruff CB, Ensrud K, Nevitt MC, Genant HK, Cummings SR (2001). Structural adaptation to changing skeletal load in the progression toward hip fragility: the study of osteoporotic fractures. J Bone Miner Res Off J Am Soc Bone Miner Res.

[30] Berry SD, Miller RR (2008). Falls: epidemiology, pathophysiology, and relationship to fracture. Curr Osteoporos Rep.

[31] Nordstrom A, Olsson T, Nordstrom P (2005). Bone gained from physical activity and lost through detraining: a longitudinal study in young males. Osteoporos Int.

[32] Tveit M, Rosengren BE, Nyquist F, Nilsson JA, Karlsson MK (2013). Former male elite athletes have lower incidence of fragility fractures than expected. Med Sci Sports Exerc.

[33] Gillespie LD, Robertson MC, Gillespie WJ, Sherrington C, Gates S, Clemson LM, Lamb SE (2012). Interventions for preventing falls in older people living in the community. Cochrane Database Syst Rev.

[34] Owen N, Sparling PB, Healy GN, Dunstan DW, Matthews CE (2010). Sedentary behavior: emerging evidence for a new health risk. Mayo Clin Proc.

[35] Ingle L, Sleap M, Tolfrey K (2006). The effect of a complex training and detraining programme on selected strength and power variables in early pubertal boys. J Sports Sci.

[36] Daly RM, Stenevi-Lundgren S, Linden C, Karlsson MK (2008). Muscle determinants of bone mass, geometry and strength in prepubertal girls. Med Sci Sports Exerc.

[37] Faigenbaum AD, Westcott WL, Loud RL, Long C (1999). The effects of different resistance training protocols on muscular strength and endurance development in children. Pediatrics.

[38] Kanehisa H, Kuno S, Katsuta S, Fukunaga T (2006). A 2-year follow-up study on muscle size and dynamic strength in teenage tennis players. Scand J Med Sci Sports.

[39] Lofgren B, Daly RM, Nilsson JA, Dencker M, Karlsson MK (2013). An increase in school-based physical education increases muscle strength in children. Med Sci Sports Exerc.

[40] Malina R (1978). Human Growth, Chapter in Human Growth: Postnatal growth by Frank Falkner. Growth of Muscle Tissue and Muscle Mass.

[41] Holloszy JO, Coyle EF (1984). Adaptations of skeletal muscle to endurance exercise and their metabolic consequences. J Appl Physiol Respir Environ Exerc Physiol.

[42] Marini M (2010). The exercised skeletal muscle: a review. Eur Transl Myology-Reviews.

[43] Sanchis-Moysi J, Idoate F, Serrano-Sanchez JA, Dorado C, Calbet JA (2012). Muscle hypertrophy in prepubescent tennis players: a segmentation MRI study. PLoS ONE.

[44] Blimkie CJ (1993). Resistance training during preadolescence. Issues and controversies. Sports Med.

[45] Strong WB, Malina RM, Blimkie CJ, Daniels SR, Dishman RK, Gutin B, Hergenroeder AC, Must A, Nixon PA, Pivarnik JM, Rowland T, Trost S, Trudeau F (2005). Evidence based physical activity for school-age youth. J Pediatr.

[46] Physical activity guidelines for Americans (2008). Physical activity guidelines for Americans. Okla Nurse.

[47] Baggett CD, Stevens J, McMurray RG, Evenson KR, Murray DM, Catellier DJ, He K (2008). Tracking of physical activity and inactivity in middle school girls. Med Sci Sports Exerc.

[48] Telama R, Yang X, Viikari J, Valimaki I, Wanne O, Raitakari O (2005). Physical activity from childhood to adulthood: a 21-year tracking study. Am J Prev Med.

[49] Nagano A, Komura T (2003). Longer moment arm results in smaller joint moment development, power and work outputs in fast motions. J Biomech.

[CR50] The pre-publication history for this paper can be accessed here:http://www.biomedcentral.com/1471-2474/15/353/prepub

