# Supplementary figures and images for: A 3-year school-based exercise intervention improves muscle strength - a prospective controlled population-based study in 223 children
Source: BMC Musculoskelet Disord. 2014 Oct 27;15:353. doi: 10.1186/1471-2474-15-353 (PMC4223838; doi:10.1186/1471-2474-15-353)

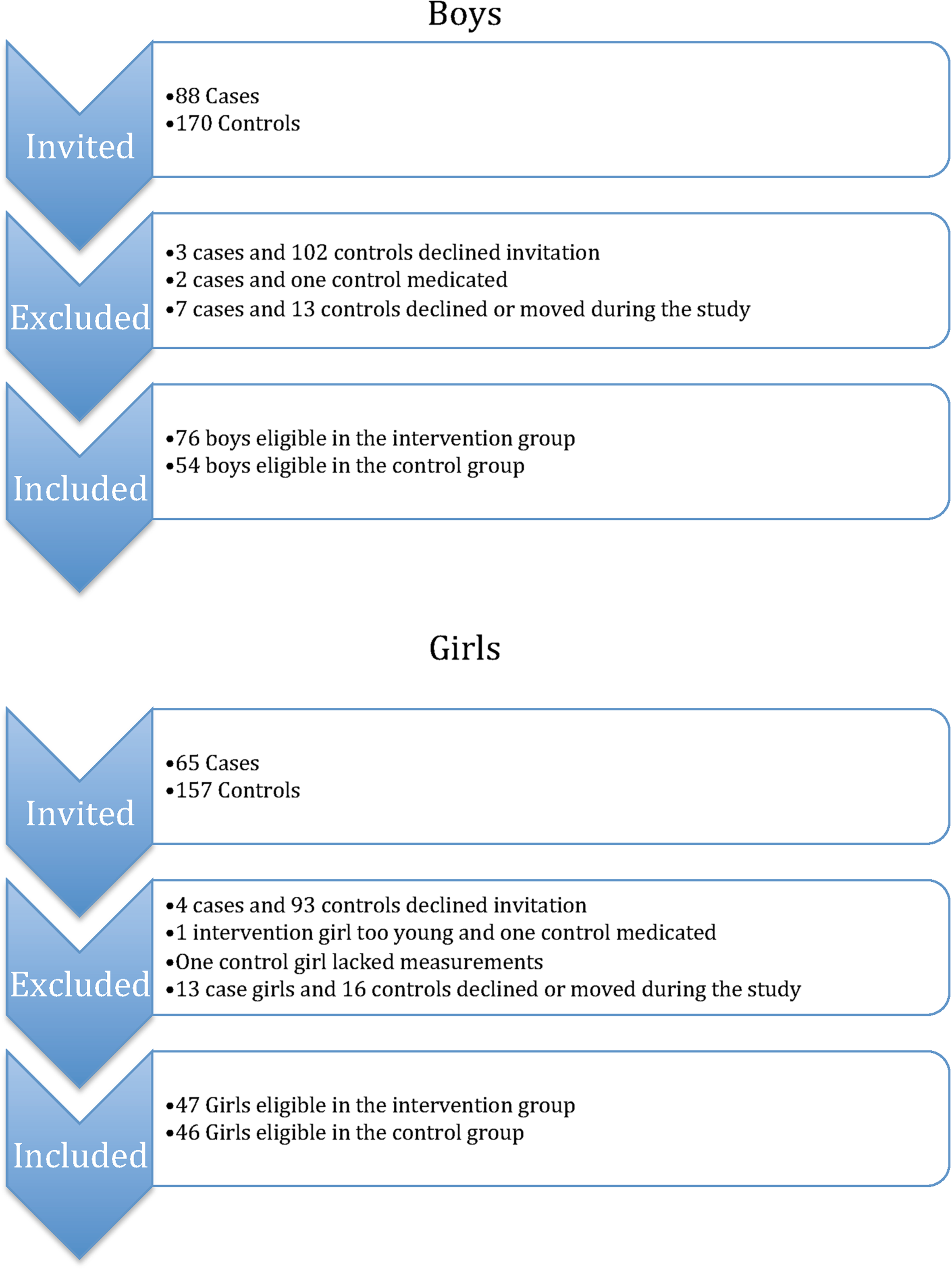

Supplement: Supplementary file 1 — Authors’ original file for figure 1 [file 12891_2014_2292_MOESM1_ESM.tif]

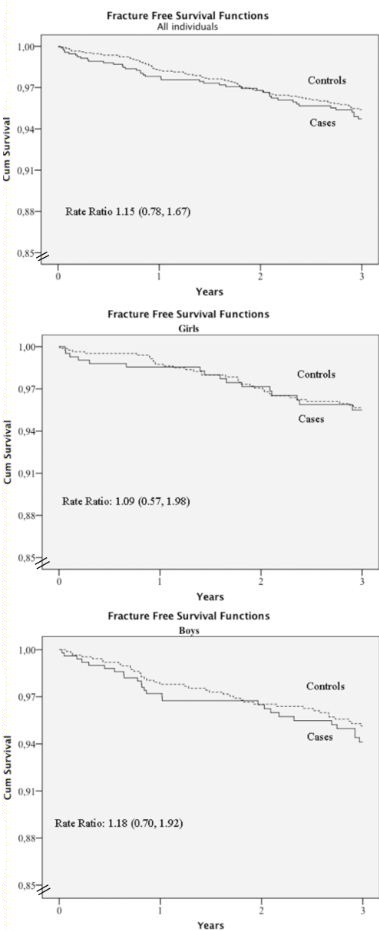

Supplement: Supplementary file 2 — Authors’ original file for figure 2 [file 12891_2014_2292_MOESM2_ESM.jpeg]
